# Chromium (VI) in phosphorus fertilizers determined with the diffusive gradients in thin-films (DGT) technique

**DOI:** 10.1007/s11356-020-08761-w

**Published:** 2020-04-18

**Authors:** Christian Vogel, Marie C. Hoffmann, Oliver Krüger, Vadim Murzin, Wolfgang Caliebe, Christian Adam

**Affiliations:** 1 Bundesanstalt für Materialforschung und –prüfung (BAM), Unter den Eichen 87, 12205 Berlin, Germany; 2 Department of Chemical and Product Safety, Bundesinstitut für Risikobewertung (BfR), Max-Dohrn-Str. 8-10, 10589 Berlin, Germany; 3 DESY, Notkestrasse 85, 22603 Hamburg, Germany; 4 Bergische Universität Wuppertal, Gaußstraße 20, 42119 Wuppertal, Germany

**Keywords:** Phosphorus recovery, Sewage sludge ash, Hexavalent chromium (Cr(VI)), Chemical extraction, X-ray adsorption near-edge structure (XANES) spectroscopy

## Abstract

Phosphorus (P) fertilizers from secondary resources became increasingly important in the last years. However, these novel P-fertilizers can also contain toxic pollutants such as chromium in its hexavalent state (Cr(VI)). This hazardous form of chromium is therefore regulated with low limit values for agricultural products even though the correct determination of Cr(VI) in these fertilizers may be hampered by redox processes, leading to false results. Thus, we applied the novel diffusive gradients in thin-films (DGT) technique for Cr(VI) in fertilizers and compared the results with the standard wet chemical extraction method (German norm DIN EN 15192) and Cr K-edge X-ray absorption near-edge structure (XANES) spectroscopy. We determined an overall good correlation between the wet chemical extraction and the DGT method. DGT was very sensitive and for most tested materials selective for the analysis of Cr(VI) in P-fertilizers. However, hardly soluble Cr(VI) compounds cannot be detected with the DGT method since only mobile Cr(VI) is analyzed. Furthermore, Cr K-edge XANES spectroscopy showed that the DGT binding layer also adsorbs small amounts of mobile Cr(III) so that Cr(VI) values are overestimated. Since certain types of the P-fertilizers contain mobile Cr(III) or partly immobile Cr(VI), it is necessary to optimize the DGT binding layers to avoid aforementioned over- or underestimation. Furthermore, our investigations showed that the Cr K-edge XANES spectroscopy technique is unsuitable to determine small amounts of Cr(VI) in fertilizers (below approx. 1% of Cr(VI) in relation to total Cr).

**Electronic supplementary material:**

The online version of this article (10.1007/s11356-020-08761-w) contains supplementary material, which is available to authorized users.

## Introduction

Phosphorus (P) is essential for all living beings and is supplied by their respective nutrition. It is removed from farmlands in the form of agricultural products and must be replaced by application of fertilizers to allow continuous farming. Since phosphate rock and P became a scarce resource in the European Union (EU 2017), several processes are under development to recover P from wastewater for fertilizer production (Schaum 2018; Kratz et al. 2019). However, these novel P-fertilizers can also contain pollutants that are toxic for plants, animals, and humans.

One potential pollutant in P-fertilizers is chromium (Cr) in the hexavalent oxidation state (Cr(VI)) also called chromate (CrO_4_^2−^) or dichromate (Cr_2_O_7_^2−^). In contrast to the trivalent form Cr(III), which is an essential trace element, Cr(VI) is a highly mobile, toxic, and carcinogenic compound (Shahid et al. 2017). Therefore, the German fertilizer ordinance (2012) has a strict limit of 2 mg kg^−1^ Cr(VI) in fertilizers. Previous research (Krüger et al. 2017) revealed that various kinds of common and novel fertilizers can contain Cr(VI). An established chemical extraction method for the determination of Cr(VI) is the German standard DIN EN ISO 15192. In this method, an alkaline media is used at temperatures above 90 °C to extract Cr(VI) and subsequently determine its concentration. However, the use of elevated temperatures and high pH values may hamper the correct determination of Cr(VI) concentrations especially in organic- and/or Fe(II)-rich matrices. The organic matter and Fe(II) in these matrices can reduce Cr(VI) and therefore cause misleading results. To overcome this problem, this study applied the novel diffusive gradients in thin-films (DGT) technique where water is used to extract the Cr(VI) from the solid material at ambient temperature. Consequently, the transformation of Cr species by high pH values at high temperatures is avoided by the DGT method. The DGT approach was subsequently compared with the aforementioned standard extraction method and observations made with Cr K-edge X-ray absorption near-edge structure (XANES) spectroscopy.

Previous DGT studies showed that the amount of nutrients and pollutants adsorbed by the binding layer of the DGT method nicely correlates to their bioavailability (Davison 2016; Vogel et al. 2017). The DGT device consists of a binding layer, a diffusion gel, and a filter (to protect the gel) assembled in a plastic holder (Davison 2016). The dissolved and labile Cr(VI) fraction of the fertilizer from moist fertilizer samples diffuses through the filter and the diffusion gel. During deployment, it subsequently adsorbs on a Cr(VI) selective binding layer (Pan et al. 2015). However, small amounts of Cr(III) may also adsorb to that binding layer. Hence, to evaluate the Cr(VI) selectivity of the DGT method, we applied Cr K-edge XANES spectroscopy because this approach enables a clear differentiation between the two oxidation states. In fact, a compound containing Cr(VI) will exhibit a prominent pre-peak within the XANES spectra, whereas compounds without any hexavalent Cr will not show this feature (Vogel et al. 2014, 2015a.

## Materials and methods

### Fertilizers origin and treatment

To provide samples with a variety of different Cr(VI) mass fractions, a recycling P-fertilizer was chosen as a model substance for this investigation. The recycling P-fertilizers are produced from sewage sludge ashes by thermochemical treatment with a sodium additive to produce the bioavailable phosphate form CaNaPO_4_ (Stemann et al. 2015; Herzel et al. 2016). In contrast to the real thermochemical process, which is generally operated under reducing conditions, the samples here were prepared under oxidizing conditions to trigger Cr(VI) formation. The SSA originated from industrial-fluidized bed incinerators in Germany, where sewage sludge from municipal wastewater treatment plants was incinerated solely. This SSA was thermochemically treated with different amounts of Na_2_CO_3_ to prepare the Cr(VI) containing model substances for the investigation. Therefore, 40 g of SSA was mixed with 1 g (SSA-Na1), 4 g (SSA-Na2), 7 g (SSA-Na3), 10 g (SSA-Na4), 11 g (SSA-Na5; similar to P-fertilizer in Vogel et al. 2014 and Krüger et al. 2017), and 20 g (SSA-Na6) of Na_2_CO_3_ (Fisher Chemicals Loughborough, UK), respectively. Afterwards, it was thermochemically treated at 950 °C for 30 min, under oxidizing conditions (air) in a muffle furnace (Nabertherm LH 15/14, Lillenthal, Germany).

Furthermore, a sedimentary phosphate rock (PR) from Morocco, a triple super phosphate (TSP) and a converter lime (CL) were used as potentially Cr(VI) bearing fertilizers for the investigation.

All fertilizer samples were air-dried, divided representatively by a dividing cross, and grinded with a tungsten carbide vibratory disc mill (see also Krüger et al. 2017). The total Cr amount of the fertilizers was measured by ICP-OES (Thermo iCAP 7000 Series, Dreieich, Germany) after total digestion (HNO_3_/HClO_4_/HF) in a microwave (mikroPrepA, MLS GmbH, Leutkirch, Germany; heating with 1000 W; 20 min isotherm segment at 240 °C). These measurements were carried out in triplicate.

### Wet chemical chromium(VI) extraction (DIN method)

We performed Cr(VI) wet chemical extraction according to DIN EN 15192 (2006). The procedure consists of an alkaline extraction of the solid sample, followed by filtration for liquid-solid separation and measurement of Cr(VI) by ICP-MS (Thermo iCAP Q, Dreieich, Germany). Therefore, 2.5 g of the sample (weighed in on 0.1 mg) were put in a 250 mL beaker, and the following chemicals were added: 50 mL of the extraction solution containing 0.5 mol L^−1^ NaOH and 0.28 mol L^−1^ Na_2_CO_3_ (Fisher Chemicals Loughborough, UK), 1 mL of 4.2 mol L^−1^ MgCl_2_∙6H_2_O (Merck, Darmstadt, Germany) to avoid Cr(III) oxidation by oxygen, and a buffer solution of each 0.5 mol L^−1^ K_2_HPO_4_ and KH_2_PO_4_ (both Carl Roth, Karlsruhe, Germany). We capped the beaker with a watch glass and heated the solution for 1 h at 92.5 °C on a magnetic hot plate stirrer with a PTFE-coated magnetic stir bar. The temperature was controlled with a contact thermometer to avoid boiling of the solution as well as evaporation to dryness. The measurements were carried out in triplicate.

### Chromium(VI) DGT extraction

The content of mobile Cr(VI) in the various fertilizers was also analyzed by DGT devices (window size, 2.54 cm^2^) equipped with a 0.8 mm APA (polyacrylamide) diffusion layer and a 0.6 mm Cr(VI) selective N-methyl-D-glucamine (NMDG) binding layer (Pan et al. 2015; purchased from DGT Research, Lancaster, UK). After a 24 h conditioning period of the fertilizer at 60% of the water holding capacity (WHC; ISO 2012), the fertilizers were brought to 100% WHC, transferred onto the DGT devices, and deployed for 24 h at 25 °C. Subsequently, the extraction of adsorbed Cr from the DGT binding layer was carried out with 1 M HNO_3_ for 24 h. Afterwards, the Cr concentrations of the extracts were analyzed by ICP-MS (Thermo iCAP Q, Dreieich, Germany) and used to calculate the DGT values. The C_DGT_ values of Cr(VI) were calculated using the following equation:

1 $$ {C}_{\mathrm{DGT}}=\frac{M\Delta  \mathrm{g}}{DAt} $$

where *M* is the mass of Cr(VI) accumulated on the binding gel, Δg is the diffusion gel thickness, *D* is the diffusion coefficient of Cr(VI) for the deployment temperature, *A* is the area of the exposure window of the DGT device, and *t* the deployment time. The diffusion coefficient for Cr(VI) at 25 °C is 8.82 × 10^−6^ cm^2^ s^−1^ (Pan et al. 2015). The DGT measurements were carried out in triplicate.

Furthermore, DGT devices (window size, 2.54 cm^2^) with 0.8 mm APA diffusion layer and 0.6 mm NMDG binding layer (DGT Research, Lancaster, UK) were loaded with 200 mL solutions (30 mg Cr L^−1^) of various water-soluble Cr(III) (CrCl_3_) and Cr(VI) (NaCrO_4_, K_2_CrO_4_, K_2_CrO_7_) compounds. The DGT devices were deployed for 24 h at 25 °C in constantly agitated solutions. After deployment, the binding layers of the DGT devices were washed with distilled water, dried at room temperature, and analyzed by Cr K-edge XANES spectroscopy (see below).

### Chromium references for Cr K-edge XANES spectroscopy

The following compounds were used for the chromium K-edge XANES spectroscopy measurements: CaCrO_4_, Cr_2_(SO_4_)_3_·H_2_O, Cr_2_S_3_ (all ABCR, Karlsruhe, Germany), CrPO_4_·4H_2_O (Alfa Aesar, Karlsruhe, Germany), Cr_2_O_3_, CrCl_3_·6H_2_O, K_2_Cr_2_O_7_ (all Merck, Darmstadt, Germany), K_2_CrO_4_ (AppliChem, Darmstadt, Germany), and Na_2_CrO_4_ (Acros, Geel, Belgium). Cr(OH)_3_ was precipitated from an aqueous solution of chromium chloride (CrCl_3_; p.a., Sigma-Aldrich, Australia) with ammonia. CaCr_2_O_4,_ MgCr_2_O_4_, and FeCr_2_O_4_ were prepared from Cr_2_O_3_ (p.a., Merck, Darmstadt, Germany) with calcium carbonate (CaCO_3_; Sigma-Aldrich, Steinheim, Germany), magnesium carbonate (MgCO_3_; Merck, Darmstadt, Germany), and Fe_3_O_4_ (ABCR, Karlsruhe, Germany) at 1250 °C, 1000 °C, and 1500 °C, respectively, in platinum crucibles by thermal treatment (6–16 h) in a muffle furnace (Nabertherm LH 15/14, Lillenthal, Germany). Cr-substituted FeOOH was prepared after Frommer et al. (2009).

### Chromium K-edge XANES spectroscopy of fertilizers

Chromium K-edge XANES spectroscopy measurements were carried out at P64 beamline (Caliebe et al. 2019) at the electron storage ring PETRAIII (DESY, Hamburg, Germany). The incident beam was monochromatized with a Si<111> double crystal monochromator. The scans were acquired at 50 K, to reduce radiation damage in the sample. The Cr K-edge XANES spectra were collected in the range of 5980–6100 eV with a Canberra 100 pixel high-purity germanium detector in fluorescence mode for the fertilizer samples. The Cr references were measured in transmission mode using two ion chambers filled with nitrogen. All spectra were background subtracted and normalized to an edge jump of Δμd = 1.

Afterwards, Cr K-edge XANES spectra of the fertilizers were fitted with linear combination fitting (LCF) of Cr reference compounds (following mentioned as XANES-LCF) with the software Demeter Athena (Ravel and Newville 2005). Therefore, the following XANES spectra were used: CaCrO_4_, Na_2_CrO_4_, K_2_CrO_4_, K_2_Cr_2_O_7_, Cr_2_O_3_, MgCr_2_O_4_, CaCr_2_O_4_, FeCr_2_O_4_, CrCl_3_, Cr(OH)_3_, CrPO_4_, and Cr-sub. FeOOH. The relative proportions of the components, whose number was limited to three, were forced to add up to 100%. From the remaining fits, the best fit was chosen, as seen by the highest *R* value.

A second method, in the following named XANES-H, was applied to calculate the Cr(VI) amounts from the XANES spectra for comparison. Here, the height of the pre-peak was analyzed in relation to the height of the edge:

2 $$ \mathrm{XANES}-\mathrm{H}=\frac{\mathrm{Height}\ \mathrm{pre}-\mathrm{peak}\ \left(5992.8\ \mathrm{eV}\right)-\mathrm{H}\mathrm{eight}\ \mathrm{baseline}\ \left(5990\ \mathrm{eV}\right)}{\mathrm{Height}\ \mathrm{edge}\ \left(6009\ \mathrm{eV}\right)-\mathrm{H}\mathrm{eight}\ \mathrm{baseline}\ \left(5990\ \mathrm{eV}\right)} $$

The values from the reference spectra of Cr_2_O_3_ and CaCrO_4_ were set to 0% and 100% Cr(VI), respectively.

## Results and discussion

### Chromium and Cr(VI) contents of the investigated fertilizers

In Table 1, the amounts of total Cr and Cr(VI) are listed for the different P-fertilizers. For Cr(VI), the data of the different investigated methods are given, respectively. For the series of thermochemically treated SSA (SSA, SSA-Na1 – SSA-Na6), it can be clearly seen that the total Cr content decreases from 118.3 (SSA) to 86.9 mg/kg (SSA-Na6) due to the dilution with increasing amounts of added Na_2_CO_3_. At the same time, the formation of Cr(VI) is clearly initiated by thermochemical reaction with the Na additive and oxygen starting from 0.2 Cr(VI) in SSA-Na3 and increasing to 68.5 mg/kg Cr(VI) present in SSA-Na6 (data of DIN method) (see also Fig. S1). A thermochemical treatment according to recipe SSA-Na4 would lead to Cr(VI) mass fractions above the limit of 2 mg/kg of the German Fertilizer Ordinance. The tested TSP and the PR have both Cr(VI) mass fractions below the limit of 2 mg/kg (DIN method). The converter lime CL showed with 7.4 mg/kg Cr(VI) a value above the limit for fertilizers in Germany (DIN method).

**Table 1:** Total Cr mass fraction and Cr(VI) amount (with standard deviation) analyzed with the wet chemical extraction (DIN), DGT, and Cr K-edge XANES spectroscopy (number in brackets is ratio Cr(VI)/total Cr) method, respectively, for various fertilizers

	Total Cr	Cr(VI)			
		DIN	DGT	XANES-LCF	XANES-H
	mg/kg	mg/kg	μg/L	mg/kg	mg/kg
SSA	118.3 ± 4.6	0.1 ± 0.1 (0%)	0.3 ± 0.1	bql	bql
SSA-Na1	113.9 ± 1.3	0.1 ± 0.1 (0%)	0.2 ± 0.1	bql	bql
SSA-Na2	110.6 ± 3.7	0.1 ± 0.1 (0%)	bql	bql	bql
SSA-Na3	102.3 ± 2.2	0.2 ± 0.1 (0.2%)	14.3 ± 1.2	bql	0.8 (0.8%)
SSA-Na4	98.1 ± 0.4	8.1 ± 0.1 (8%)	84.2 ± 1.5	11.1 (11%)	6.8 (7%)
SSA-Na5	95.1 ± 1.5	12.3 ± 0.2 (13%)	110.2 ± 2.2	19.3 (20%)	10.0 (11%)
SSA-Na6	86.9 ± 6.1	68.5 ± 0.2 (78%)	276.5 ± 3.6	50.8 (58%)	36.7 (42%)
PR	177.0 ± 2.1	0.7 ± 0.2 (0.4%)	11.2 ± 2.1	bql	bql
TSP	77.6 ± 1.4	0.5 ± 0.1 (0.6%)	36.6 ± 2.8	bql	bql
CL	1168.5 ± 13.6	7.4 ± 0.2 (0.6%)	7.6 ± 1.1	bql	bql

*bql* below quantification limit, *PR* phosphate rock, *TSP* triple superphosphate, *CL* converter lime

### Applicability of the DGT method for the determination of Cr(VI) in P-fertilizers

The DGT method adsorbs mobile Cr(VI) from the fertilizer/water mixture for a certain definite time span and is thus not an absolute method. The data from DGT are given in μg L^−1^ after extraction of the DGT binding layer following a defined procedure (see “Materials and methods” section). Thus, a calibration with standard materials would be required to determine Cr(VI) mass fractions by DGT which was not part of this work. However, we found a good correlation (*R*^2^ = 0.93) between the wet chemical extraction (DIN method) and the data determined by the DGT method for the series of ash-based model fertilizers SSA – SSA-Na6 (see Fig. S2).

**Fig. 1:**
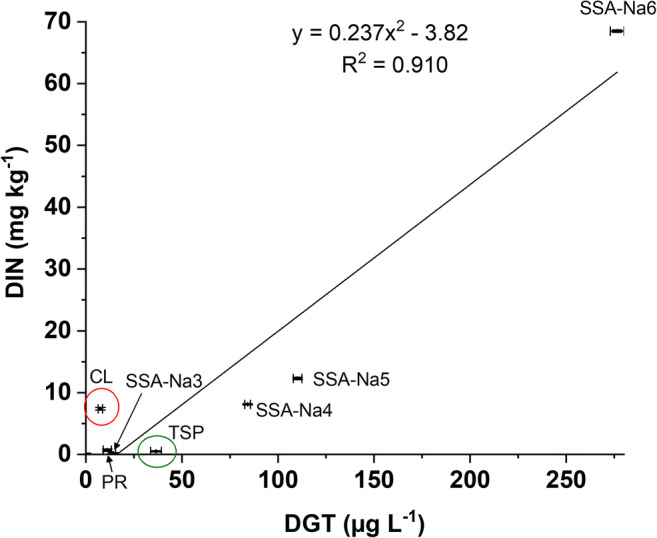
Comparison of Cr(VI) mass fractions in fertilizers between DIN (wet chemical) method and DGT method (values with standard deviation). The red circle shows CL, and the green circle shows TSP. The points of the fertilizers SSA to SSA-Na2 are at the zero intersection

However, the conventional fertilizers TSP and CL show no correlation between DIN and DGT results (see red and green circle, respectively, in Fig. 1). The CL fertilizer contains a very high mass fraction of CaO (> 40%) which could support the development of CaCrO_4_ (Vogel et al. 2015b). This Cr(VI) compound is, in contrast to the other Cr(VI) compounds, only hardly water-soluble (Vogel et al. 2014) which might be the reason for the low DGT value. In contrast, TSP has a much higher DGT value compared with wet chemical extraction. TSP is made from phosphate rock which is treated with phosphoric acid during production. Here, a formation of mobile Cr(III) species could be possible. In fact, Pan et al. (2015) stated that small amounts of Cr(III) can also be adsorbed to the DGT binding layer. Thus, to determine the Cr(VI) selectivity and robustness of the DGT method, the investigated P-fertilizers and their DGT binding layers were analyzed by Cr K-edge XANES spectroscopy after deployment.

### Comparison between DGT method and XANES spectroscopy

**Fig. 2:**
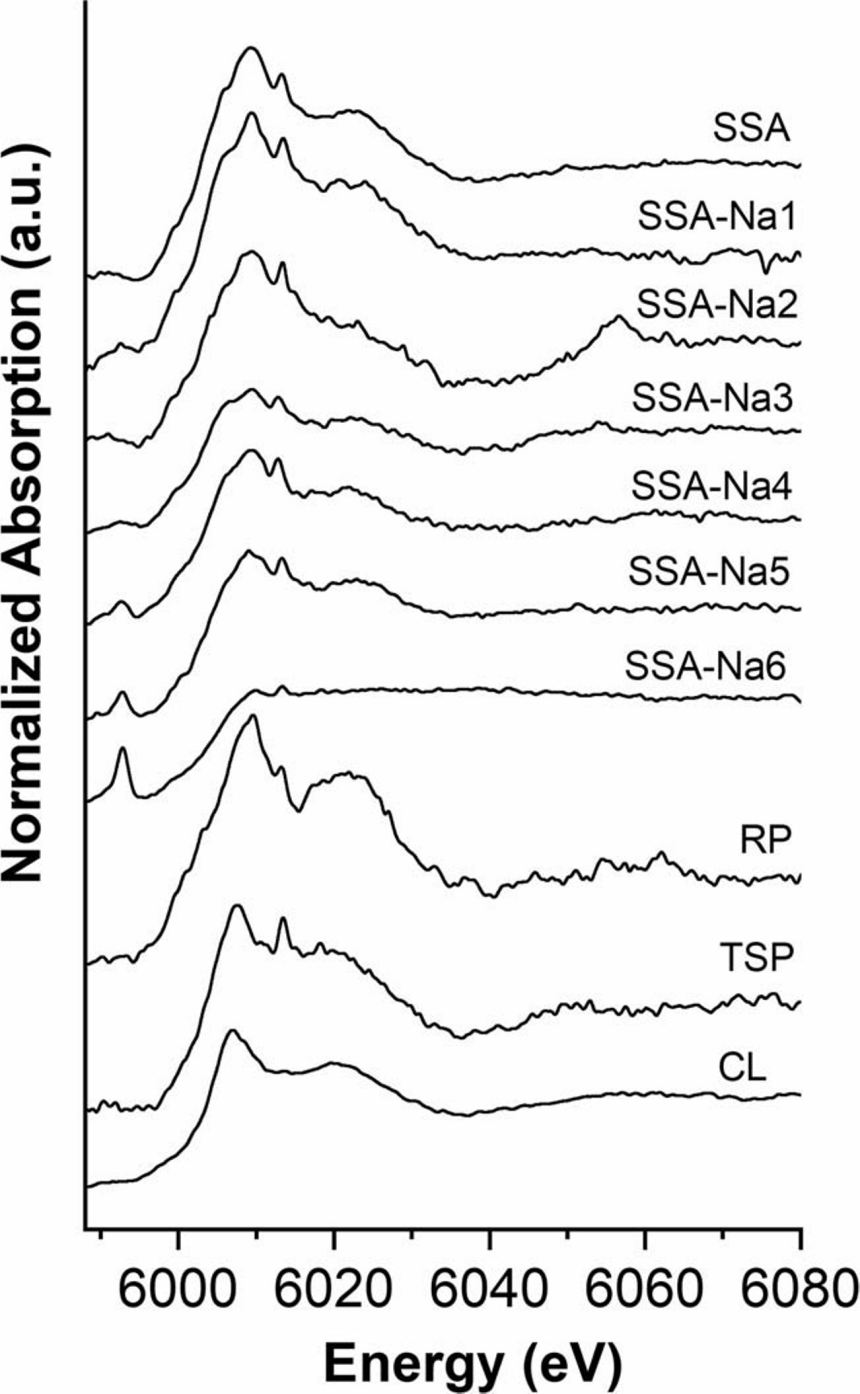
Chromium K-edge XANES spectra of various fertilizers

**Fig. 3:**
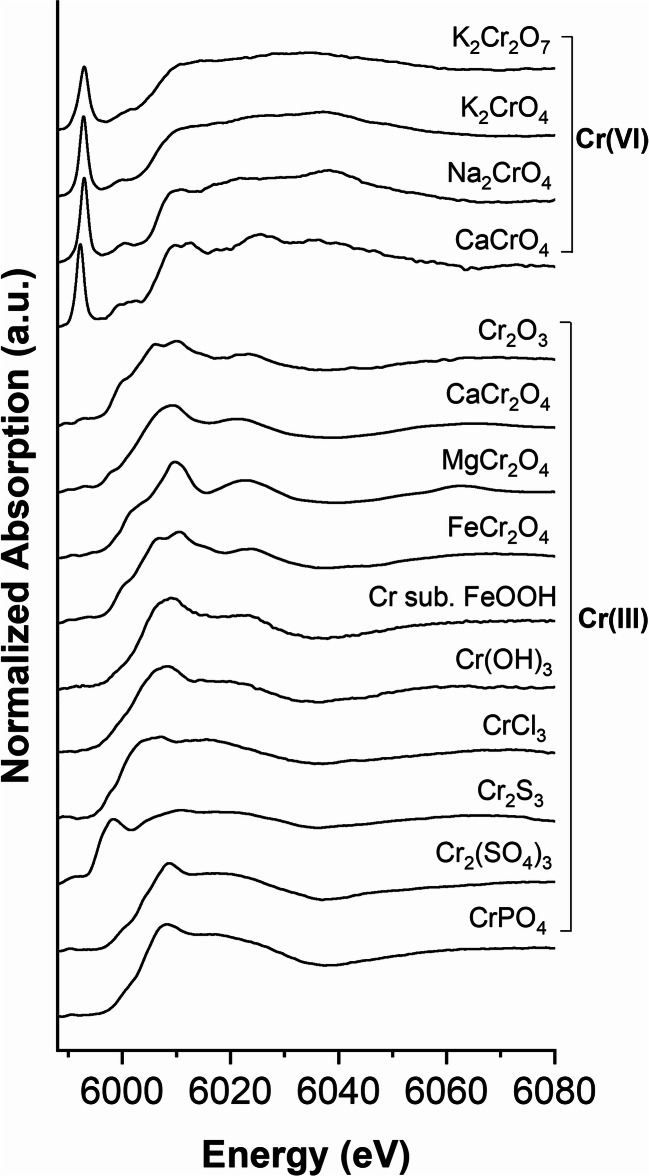
Chromium K-edge XANES spectra of various Cr compounds

**Fig. 4:**
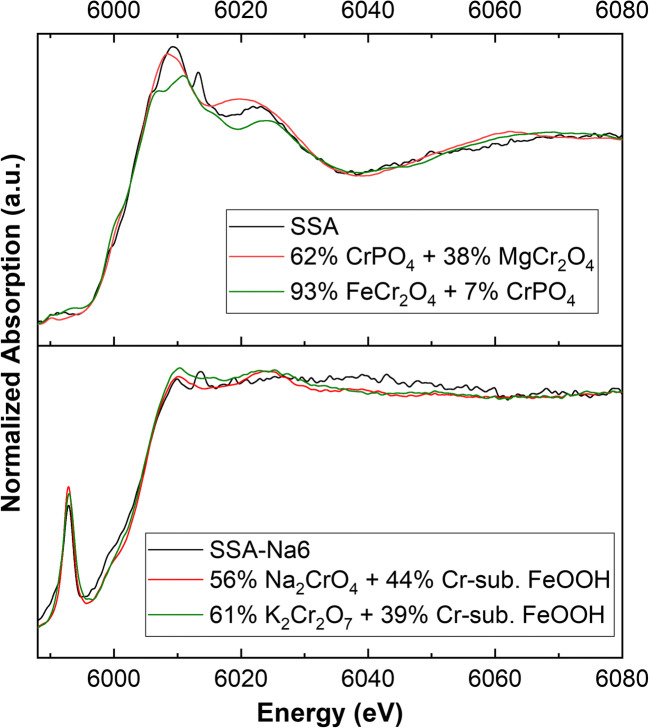
Chromium K-edge XANES spectrum of SSA (top) and SSA-Na6 (bottom) and corresponding linear combination fittings of different Cr references

Figure 2 shows the Cr K-edge XANES spectra of various P-fertilizers. All spectra have a white line at 6008 eV and a post-edge feature at 6023 eV (except for SSA-Na6). These features come close to those of Cr(III) reference compounds presented in Fig. 3. The fertilizers SSA-Na4, SSA-Na5, and SSA-Na6 show an additional pre-peak of Cr(VI) at 5992 eV. Linear combination fitting (LCF) of SSA (Fig. 4 top) indicates mainly chromite (MgCr_2_O_4_ or FeCr_2_O_4_) and chromium phosphate as Cr-bearing phases. Very similar results were also detected for the materials SSA-Na1 to SSA-Na3. In contrast, the LCF fit of SSA-Na6 (Fig. 4 bottom) also indicates Cr(VI) compounds in the fertilizer. A similar result was found for SSA-Na4 and SSA-Na5. From the results of the LCF fits and the total mass fractions of Cr (Table 1) in the fertilizers, the Cr(VI) amount was calculated (see XANES-LCF in Table 1). In contrast to the LCF of the XANES spectra (XANES-LCF), the DGT and wet chemical Cr(VI) analyses indicate also Cr(VI) in SSA-Na3, PR, TSP, and CL (Table 1). Only the results from the SSA-based materials with higher Cr(VI)-contents show a good correlation to the DGT method (*R*^2^ = 0.99; Fig. S3).

In contrast to the XANES-LCF approach, the XANES-H approach indicates Cr(VI) in SSA-Na3 to SSA-Na6 as it was observed by DGT and the wet chemical DIN method. Similarly to the XANES-LCF method, there is a good correlation between the XANES-H and DGT method for SSA-based fertilizers (*R*^2^ = 0.97; Fig. S4) and an even better agreement for SSA-Na3. For the RP, TSP, and CL fertilizers, there is no agreement between DGT, wet chemical, and XANES-H method, since the latter indicates no Cr(VI) in these samples. Similarly to our approach, Bajt et al. (1993) and Rinehart et al. (1997) used a method where the area of the pre-peak was calculated. These authors revealed that fractions of 5% and 3% Cr(VI) out of total Cr could barely be detected by Cr K-edge XANES spectroscopy, respectively. It is therefore likely to assume that a fraction of 3% Cr(VI) out of total Cr represents the quantification limit for this technique. However, this XANES Cr(VI) quantification limit is independent from the total Cr amount. RP, TSP, and CL contain only 0.4–0.6% Cr(VI) in relation to total Cr (according to DIN method; Table 1) and thus much less than the proposed quantification limit. Accordingly, these samples could be below the quantification limit of Cr K-edge XANES spectroscopy. In contrast, SSA-Na3 has only a Cr(VI) amount of 0.2% in relation to total Cr (DIN method, Table 1), but with the XANES-H method, a little bit higher amount (0.8%, Table 1) could be detected. Thus, there might be a possibility that a quantification with the XANES-H method might be possible down to approx. 1% Cr(VI) in relation to total Cr.

### XANES spectroscopy investigations of deployed DGT binding layers

**Fig. 5:**
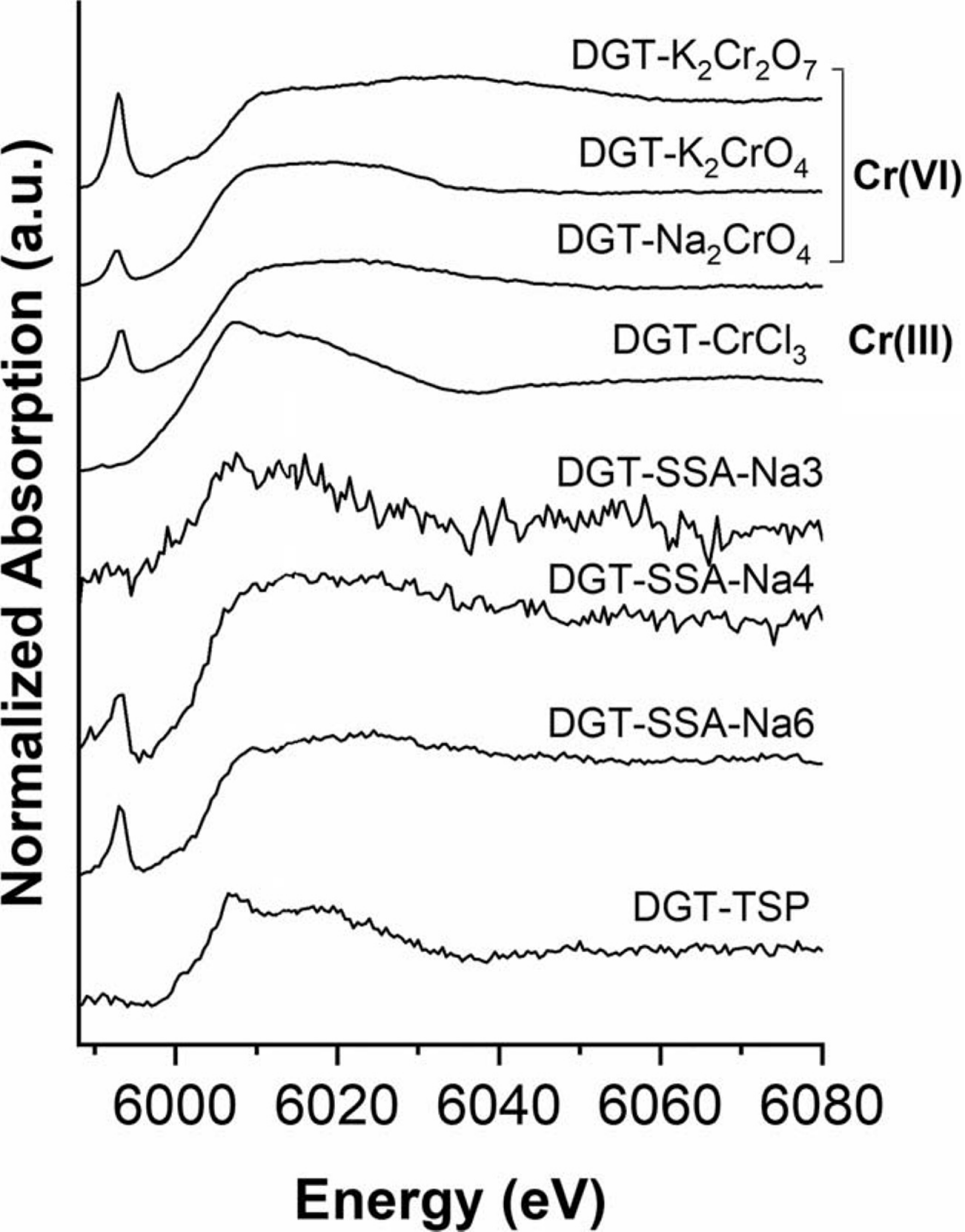
Chromium K-edge XANES spectra of various deployed DGT binding layers

Figure 5 (top) shows the Cr K-edge XANES spectra of the Cr(VI) selective DGT binding layers saturated with various Cr(III) and Cr(VI) compounds. The XANES spectra of the Cr(VI) saturated binding layers (top) are very similar to those of solid Cr(VI) compounds (see Fig. 3) and also show the characteristic pre-peak. In contrast, the Cr(III) saturated binding layer (DGT-CrCl_3_) has no pre-peak, and the XANES spectrum features are close to those of solid chromites (see Fig. 3).

Due to the very low amounts of adsorbed Cr on the DGT binding layer, it was not possible to collect Cr K-edge XANES spectra for all deployed binding layers of the various fertilizers. SSA-Na4 and SSA-Na6 (SSA-Na5 was not investigated) show the Cr(VI) pre-peak, and the spectra come close to those of the Cr(VI) DGT references. Due to the high signal-to-noise ratio of the XANES spectrum of SSA-Na3, a detection of a Cr(VI) pre-peak was impossible (see Fig. 5). In contrast to SSA-Na4 and SSA-Na6, the XANES spectrum of the deployed binding layer of TSP did not show a pre-peak. Furthermore, this XANES spectrum is also very similar to that of the DGT-Cr(III) reference. Therefore, we assume that TSP contains mobile Cr(III) that adsorbs also on the Cr(VI) selective binding layer. Thus, the existence of mobile Cr(III), which probably develops during the production of TSP, where phosphate rock is treated with phosphoric acid, might lead to an overestimation of Cr(VI) with the DGT method.

## Conclusion

All three investigated methods for the determination of Cr(VI) in P-fertilizers from secondary or primary raw materials seem to have advantages and drawbacks. Krüger et al. (2017) mentioned earlier that the presence of organic matter and inorganic substances with potential to reduce Cr(VI) during wet chemical extraction according to the DIN method can cause underestimation of Cr(VI) in P-fertilizers. The DGT method was very sensitive and for most tested materials selective for the analysis of Cr(VI) in P-fertilizers made from recycled materials. However, the results of certain types of P-fertilizers containing mobile Cr(III) or hardly soluble Cr(VI) show that some optimization of the method is still required to avoid over- or underestimation of Cr(VI). Therefore, the DGT binding layer should be optimized so that mobile Cr(III) (like from fertilizer TSP) will no longer be adsorbed. Furthermore, the DGT method cannot detect hardly soluble Cr(VI). This, however, is also an advantage of this method because only the mobile and bioavailable Cr(VI), which is an important parameter for plant growth, will be analyzed. Additionally, the Cr K-edge XANES spectroscopy method also shows good results but fails to determine small amounts of Cr(VI) (below approx. 1% Cr(VI) in relation to total Cr) in fertilizers.

## Electronic supplementary material

ESM 1 (DOCX 39 kb)
