# Supplementary material for: Chromium (VI) in phosphorus fertilizers determined with the diffusive gradients in thin-films (DGT) technique
Source: Environ Sci Pollut Res Int. 2020 Apr 18;27(19):24320–8. doi: 10.1007/s11356-020-08761-w (PMC7326810; doi:10.1007/s11356-020-08761-w)
Supplement: Supplementary file 1 — (DOCX 39 kb) [file 11356_2020_8761_MOESM1_ESM.docx]

**Supplementary Material:**

*Figure S1: Correlation of Cr(VI) mass fraction (DIN method) of SSA based P-fertilizers to added amount of Na_2_CO_3_ during thermal treatment*


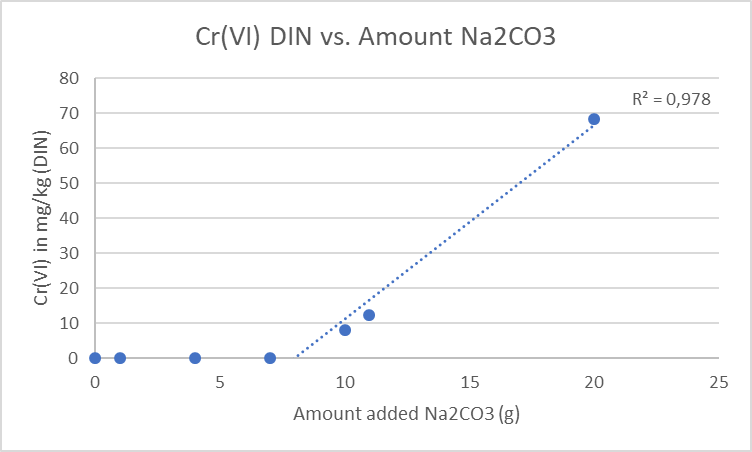


*Figure S2: Correlation of determined Cr(VI) amounts of SSA based P-fertilizers analyzed by the DGT and wet chemical (DIN) method*

*Figure S3: Correlation of determined Cr(VI) amounts of SSA based P-fertilizers analyzed by DGT and XANES-LCF method*

*Figure S4: Correlation of determined Cr(VI) amounts of SSA based P-fertilizers analyzed by the DGT and XANES-H method*
